# Anti-Hyperuricemic, Anti-Inflammatory and Analgesic Effects of *Siegesbeckia orientalis* L. Resulting from the Fraction with High Phenolic Content

**DOI:** 10.1186/s12906-017-1698-z

**Published:** 2017-04-04

**Authors:** Thuy Duong Nguyen, Phuong Thien Thuong, In Hyun Hwang, Thi Kim Huyen Hoang, Minh Khoi Nguyen, Hoang Anh Nguyen, MinKyun Na

**Affiliations:** 1 Department of Pharmacology, Hanoi University of Pharmacy, 13 Le Thanh Tong, Hoan Kiem, Hanoi, Vietnam; 2 Department of Pharmaceutical Analysis and Standardization, National Institute of Medicinal Materials, 3B Quang Trung, Hoan Kiem, Hanoi, Vietnam; 3 College of Pharmacy, Woosuk University, Wanju, Jeonbuk, 55338 Republic of Korea; 4 College of Pharmacy, Chungnam National University, Yuseong-gu, Daejeon, 34134 Republic of Korea

**Keywords:** *Siegesbeckia orientalis*, Anti-hyperuricemic activity, Anti-inflammatory activity, Analgesic activity, Xanthine oxidase, Caffeic acid analogues, Flavonones

## Abstract

**Background:**

The medicinal plant *Siegesbeckia orientalis* L. has been commonly used for the treatment of acute arthritis, rheumatism, and gout in Vietnam. However, pharmacological research of this plant associated with gout has not been reported. Anti-hyperuricemic and anti-inflammatory effects were evaluated and observed for the crude ethanol extract (CEE) of *S. orientalis*. Retention of these biological properties was found in a *n*-butanol-soluble fraction (BuOH fr.) of the extract, and therefore further biological and chemical investigations were undertaken on the BuOH fr. to support the medical relevance of this plant.

**Methods:**

The aerial part of *S. orientalis* was obtained in the mountainous region of Vietnam. The crude ethanol extract (CEE) and its BuOH fr. were prepared from the plant materials. Anti-hyperuricemic activities of the CEE and BuOH fr. were tested in vivo using the model oxonate-induced hyperuricemia rats through determination of serum uric acid levels and inhibitory effects on xanthine oxidase (XO) in the rat liver. Anti-inflammatory activities of the BuOH fr. were also evaluated in vivo using carrageenan-induced paw edema and urate-induced synovitis in rats. Active components of the BuOH fr. were characterized by comparison of HPLC retention time (*t*_R_) and spectroscopic data (UV, ^1^H–NMR) with those of reference compounds.

**Results:**

The CEE of *S. orientalis* displayed anti-hyperuricemic activity, and the BuOH fr. was found to be the most active portion of the extract. Further in vivo studies on this fraction showed 31.4% decrease of serum uric acid levels, 32.7% inhibition of xanthine oxidase (XO), 30.4% reduction of paw edema volume, symptomatic relief in urate-induced synovitis and significant analgesic effect at the dose of 120 mg/kg, as compared to the corresponding values of the control groups. Chemical analysis of the BuOH fr. revealed high phenolic content, identified as caffeic acid analogues and flavonones.

**Conclusions:**

This study suggested that anti-hyperuricemic and anti-inflammatory mechanism of *S. orientalis* is related to XO inhibitory effect of the phenolic components. Our findings support the use of this plant as the treatment of gout and other inflammatory diseases.

**Electronic supplementary material:**

The online version of this article (doi:10.1186/s12906-017-1698-z) contains supplementary material, which is available to authorized users.

## Background

The plant *Siegesbeckia orientalis* L. (syn. *S. glutinosa*), a member of Asteraceae, is widely distributed in Vietnam and other South-East Asian countries [1, 2]. The aerial part of this plant has been used for treating various inflammatory diseases, such as neurasthenia, insomnia, impetigo, furuncle, wound, and burn [2]. Being known as a Vietnamese indigenous medicine “hy-thiem”, this plant has been applied in a series of traditional remedies for the treatment of acute arthritis, rheumatism, inflammation, and especially gout and pain [1, 3, 4]. Although biological potential of *S. orientalis* has been previously investigated, the interest was limited to anti-inflammatory and analgesic activities of either whole herbal extract [5] or kirenol [2], which is an *ent*-pimarane type diterpene identified from this plant and commonly encountered from the same genus *Aster* [6–8]. Furthermore, a recent in vitro and in vivo study on the anti-inflammatory mechanism of *S. orientalis* demonstrated that its ethanol extract suppresses mitogen-activated protein kinases (MAPKs)- and NF-κB-dependent pathways [9]. Given that inflammatory response is a key step in the onset of gout symptoms [10], anti-inflammatory effects were thought to be responsible for traditional utilization of *S. orientalis* as a part of symptomatic treatment of this disorder.

Xanthine oxidase is an enzyme converting xanthine and hypoxanthine into uric acid. A high level of serum uric acid (hyperuricemia) is a well-known major cause of gout, and this metabolic syndrome is closely related to inflammatory responses [10]. Deposition of monosodium urate crystals in a joint could lead to an acute inflammatory pain. Phytochemical studies of *S. orientalis* identified various secondary metabolites, which include sesquiterpenoids [11, 12], diterpenoids [6–8, 13], and caffeic acid and rutin [14]. It is notable that in vitro xanthine oxidase (XO) inhibitory activities of caffeic acid and its analogues were reported previously [14–16], while rutin exhibited the anti-hyperuricemic effect in mice mediated by XO inhibition in vivo, but not in vitro [17, 18]. Our preliminary screening also confirmed that the ethanol extract of *S. orientalis* was a potent inhibitor of XO among more than 300 Vietnamese medicinal plants. Therefore, it was supposed that *S. orientalis* could have dual role in treatment of gout which related to both hypouricemic and anti-inflammatory activity. Based on a literature search, kirenol was suggested to be the main active compound which was responsible for the anti-inflammatory activity of *S. orientalis* [2]. To our knowledge, this compound, however, has not been observed for biological activities with regard to XO inhibition. Important active constituents involved in XO inhibition activity of *S. orientalis* therefore remain to be determined.

The present study evaluates anti-hyperuricemic and anti-inflammatory effects of *S. orientalis* extract using well-established animal models. Taking into consideration both anti-inflammatory and XO inhibitory effects, we focused on flavonoids and other phenolic compounds which are extensively studied and well-known antioxidants as potential phytochemical agents for treating diseases mediated by free radicals, including inflammation and gout [19, 20].

## Methods

### Chemicals and reagents

All the chemicals and reagents used for in vivo tests were of biological grade purchased from Sigma Aldrich (St Louis, MO, USA): xanthine 99–100% (Cat. XO626-25G; Lot#/Batch# 097 K5307), carrageenan (C1013-100G; Pcode 100,160,665); uric acid (> = 99%, crystalline, U2625); oxonic acid potassium salt (97%; 156,124-100G); xanthine oxidase, from bovine milk (X1875-50UN; 1,000,877,910). Solvents for extraction and fractionation were of industrial grade purchased from a licensed chemical company in Hanoi, Vietnam, and used without purification.

### Plant material

The aerial parts of *Siegesbeckia orientalis* L. (Asteraceae) were collected in the mountainous region of Hoa Binh province, in the North of Vietnam. The plant was authenticated by Prof. Tran Van On, Department of Botany, Hanoi University of Pharmacy. A voucher specimen (VDL-HT01) has been deposited at the Herbarium of the Department of Pharmaceutical Analysis and Standardization, Vietnam National Institute of Medicinal Materials, Hanoi, Vietnam.

### Preparation of extraction and fractionation

The dried plant material (5 kg) was extracted three times with 96% ethanol (EtOH) at room temperature for 9 days. The solution was filtered and combined, and the organic solvent was removed under reduced pressure to give a crude ethanol extract (CEE, 180 g). A part of the CEE (150 g) was suspended in water (1 L) and then successively partitioned with *n*-hexane (Hex), ethyl acetate (EtOAc), and *n*-butanol (BuOH) (each 1 L). The organic solvents and water were evaporated to yield Hex- (44 g), EtOAc- (33 g), and BuOH-soluble (30 g) fractions, and the remaining water soluble fraction (31 g).

### Animals

The adult male Wistar rats (8 weeks old, weighing 140 ± 10 g) were obtained from Animal Facilities, Research Center for Medicine and Pharmacy, Vietnam Military Medical University (Hanoi, Vietnam). Animals were used and processed according to the suggested ethical guidelines for the care of laboratory animals [21], and the experimental protocols in this study were approved by the Scientific and Ethical Committee of Hanoi University of Pharmacy (156/DHN-QD). The rats were acclimatized at least 7 days to adapt to their environment before any experimental manipulation. They were housed in 612 × 345 × 216 mm cages (Tecniplast 2000P) in an animal room (Department of Pharmacology, Hanoi University of Pharmacy) with a temperature of 24–26 °C, humidity of 55–60%, regular 12/12 h light/dark cycle (7:00 a.m. – 7:00 p.m.), and access to standard laboratory diet and tap water freely until used for experiments. General health status of the rats was monitored on alternate days, and no adverse events were recorded during the housing period. At the beginning of each experiment, body weight of the animals ranged from 180 to 220 g. All the samples from animals subjected to the treatments were included in the data analysis.

### Evaluation of anti-hyperuricemic effect

#### Drug administration

Each test sample of CEE and BuOH fraction was suspended in 0.5% sodium carboxymethylcellulose (CMC-Na). Food, but not water, was withdrawn from the animals 2 h prior to drug administration. Based on the Vietnamese traditional usage of “hy-thiem”, rats were given the CEE at 300, 600, 1200 mg/kg and the BuOH fraction at 120 mg/kg once a day for five consecutive days. Rats in the negative control group were orally administrated with 0.5% CMC-Na only, while those of the positive control group were given allopurinol at 10 mg/kg.

#### Animal hyperuricemia model

Hyperuricemia of rats was induced by potassium oxonate, an uricase inhibitor [22]. In brief, potassium oxonate was suspended in 0.5% CMC-Na. One hour before administration of the test samples, rats were intraperitoneally injected with the freshly prepared potassium oxonate suspension at the dose of 250 mg/kg to increase their serum uric acid levels. Whole blood samples were collected from the tail vein of the rats 1 h after the final administration of tested compounds. Blood was allowed to clot for approximately 1 h at room temperature and then centrifuged at 3500 rpm for 5 min to obtain the serum, which was stored at −20 °C until used.

#### Determination of blood uric acid levels

Serum uric acid levels were determined by the phosphotungstic acid method [23].

#### Enzyme preparation from rat liver

Rat liver was rapidly excised and homogenized in an ice-cold 50 mM potassium phosphate buffer (pH 8.0). The homogenate was centrifuged at 3000×*g* for 10 min at 4 °C, and then lipid layer was carefully removed. The resulting supernatant was further centrifuged at 10000×*g* for 60 min at 4 °C. The supernatant obtained from the final centrifugation was used to detect XO activity.

#### Assay for XO inhibition in rat liver

Enzyme activity of XO was assayed by monitoring uric acid formation using the spectrophotometric method as described elsewhere [24]. Briefly, a reaction was started by adding 100 μL of the supernatant to a phosphate buffer solution (pH 7.5) containing 0.12 mM xanthine and 0.192 mM EDTA. The mixture (total 5.0 mL) was incubated for 30 min at 37 °C, and the reaction was terminated by the addition of 1 M HCl (0.5 mL). The production of uric acid was determined by measuring UV absorbance at 290 nm. The XO activities were expressed as mmol of produced uric acid per minute per gram protein. Protein concentration was determined by the Lowry method [25] using bovine serum albumin as a standard.

### Evaluation of anti-inflammatory effect

#### Drug administration

Rats were randomly divided into 3 groups (*n* = 8 for each): negative control, positive control, and test sample (BuOH fraction). Indomethacin, a nonsteroidal anti-inflammatory drug, was used as a reference compound for the positive control group. Each sample of BuOH fraction and indomethacin was dissolved/suspended in 0.5% CMC-Na, and orally given to rats at the dose of 120 mg/kg and 10 mg/kg body weight, respectively. The rats in the negative control group were orally administrated with 0.5% CMC-Na only. Volume of administration was identical for all rats in three groups.

#### Carrageenan-induced rat paw edema

Paw edema test was performed according to the previously described carrageenan-induced method [26] with some modification. Briefly, 1 h after the drug administration, paw edema was induced by injecting 0.1 mL of 1% (*w*/*v*) carrageenan in buffer saline into the plantar surface of the right hind paw in all rats. The paw was marked in order to immerse it to the same extent in the measurement chamber. Volume of the rat paws was measured using a plethysmometer (Ugo Bisile, Italy) immediately before the carrageenan subplantar injection and at intervals of 1, 3, and 5 h. The assessment of paw volume was always performed in double blind and by the same operator. Indomethacin was administered p.o. as a reference drug. The increased percentage of edema was calculated as follows:

$$ \mathrm{Increased}\ \mathrm{edema}\ \left(\%\right)=\frac{Volume\  at\  the\  end\  of\ 3\  h\ (mL)}{Basal\  paw\  volume\ (mL)}\times 100 $$

The percentage inhibition of edema for each group was calculated by the following formula

$$ \mathrm{P}\%\left(\mathrm{Inhibition}\ \mathrm{of}\ \mathrm{edema}\right)=\frac{Ec- Et}{Ec}\times 100 $$

Ec = % Edema of the negative control group, and Et = % Edema of the treatment group.

#### Carrageenan-induced mechanical hyperalgesia

Mechanical hyperalgesia was examined in an inflammatory pain rat model by measuring the withdrawal thresholds of hind paw to an increasing pressure stimulus, using an analgesymeter (model 37,215; Ugo Basile, Italy). Paw withdrawal thresholds were measured in naive animals prior to the intraplantar injection of carrageenan into the hind paw. The cut-off was set at 250 g and the endpoint was taken as paw withdrawal or vocalization. Withdrawal thresholds were measured before (predose) and up to 3 h after drug or vehicle administration (postdose). In all cases, testing was done in blind. Reversal of mechanical hyperalgesia was calculated according to the following formula [27]:

$$ \%\mathrm{Reversal}=\frac{Postdose\  threshold- Predose\  threshold}{Naive\  threshold- Predose\  threshold}\times 100 $$

#### Carrageenan-induced thermal hyperalgesia

Carrageenan-induced thermal hyperalgesia was assessed in separate groups of animals. The measurement of the nociceptive response to a thermal stimulus used a hot-plate test with the temperature adjusted to 51 ± 1 °C [28]. The withdrawal latencies of the hind paw were measured. Hyperalgesia to heat was defined as a decrease in the Δ latency (sec), calculated by the difference of latency times between the carrageenan- and non-injected paw (naive). BuOH fraction was administered orally at the dose of 120 mg/kg one hour before the carrageenan injection, and the positive control group received indomethacin (10 mg/kg, p.o.).

#### Urate-induced synovitis

One hour after the drug administration, rats were anesthetized and injected with 100 μL of sodium urate crystals into one knee. The animals were allowed to walk on a metal grid after 18–24 h. A scoring system is adopted in which inflammatory symptoms ranging from tenderness, limping, occasional 3-legged gait to complete 3-legged gait are scored from 1+ to 4+ [29]. BuOH fraction was administered orally at the dose of 120 mg/kg 1 h before and 20 h after the urate injection, and the positive control group received indomethacin (10 mg/kg, p.o.).

### Statistical data analysis

Data are expressed in mean ± standard error of the mean (SEM) from eight animals per group. Statistical analysis was performed by one-way analysis of variance (ANOVA). Dunnett’s multiple range test was performed to determine significant differences among means. Kruskal-Wallis test was used to analyze non-parametric data, followed by Mann- Whitney U test if applicable. The values of *p* < 0.05 were considered to be statistically significant.

### Determination of total phenolic content

Total phenolic content was measured using the Folin-Ciocalteau method as described previously [25].

### Characterization of phenolic compounds

Phenolic compounds in the BuOH fraction were analyzed by a Shimadzu HPLC system equipped with a DAD detector and C_18_ column (250 × 4.6 mm; 5 μm), using a gradient of MeOH (solvent A) and 0.1% phosphoric acid in water (solvent B) as mobile solvents. The following HPLC method was used: flow rate (0.6 mL/min); 0–5 min (10% A); 5–7 min (10–25% A); 7–20 min (25% A); 20–40 min (25–50% A); 40–55 min (50% A); 55–63 min (50–80% A); 63–68 min (80–100% A). The compounds isolated were identified by comparison of their retention time (*t*_R_) and spectroscopic data (UV and ^1^H- and ^13^C–NMR) with those of reference compounds [25].

## Results

### Effect of CEE on serum uric acid levels in rats

The CEE at 300, 600, and 1200 mg/kg were orally administered for 5 days on oxonate-induced hyperuricemia rats, and serum uric acid levels were measured by the phosphotungstic acid method. As presented in Table 1, compared with the uric acid value of the normal rats (116.17 μmol/L), the value of the control rats (218.66 μmol/L) was significantly increased by the injection of potassium oxonate (*p* < 0.01). Administration of the CEE enabled lowering the uric acid levels of hyperuricemic rats to normal ranges at 600 and 1200 mg/kg (*p* < 0.05), while no effect was observed at dose of 300 mg/kg. The positive control, allopurinol at dose of 10 mg/kg, displayed more extensive hypouricemic activity, which significantly reduced the serum uric acid level to 101.69 μmol/L (*p* < 0.01).

**Table 1:** Inhibitory effect of crude ethanol extract (CEE) from *S. orientalis* on serum uric acid levels in rats

Group treatment	Dose (mg/kg)	Number	Serum uric acid levels (μmol/L)	Inhibition (%)
Normal			116.17 ± 8.71	
Control		8	218.66 ± 16.82^##^	
CEE	300	8	174.49 ± 12.31	20.2
	600	8	159.16 ± 16.36*	27.2
	1200	8	158.18 ± 14.07*	27.7
Allopurinol	10	8	101.69 ± 15.98**	53.5

Hyperuricemic rats were induced by a potassium oxonate injection 1 h before the last drug administration. The CEE at 300, 600 and 1200 mg/kg and allopurinol at 10 mg/kg were orally administrated once a day for five consecutive days. The control and normal groups were orally administered with 0.5% CMC-Na. The serum uric acid levels of rats were measured by the phosphotungstic acid method. Values are displayed as mean ± SEM

*p < 0.05, **p < 0.01 vs. hyperuricemic rats (control);

#*p* < 0.05, ##*p* < 0.01 vs. normal rats

### Effect of CEE and BuOH fraction on serum uric acid levels in rats

A part of CEE (150 g) was fractionated, and obtained 30 g of the BuOH-soluble portion. The CEE (600 mg/kg) and BuOH fraction (120 mg/kg) were further tested for hypouricemic activity in an animal model, and were orally administered for 5 days on oxonate-induced hyperuricemia rats, and the serum uric acid levels were measured by the phosphotungstic acid method. As presented in Table 2, the CEE and BuOH fr. of *S. orientalis* reduced uric acid levels. Of the samples tested, the BuOH fr. exhibited notable hypouricemic activity by lowering the serum uric acid level by 31.4% at dose of 120 mg/kg (*p* < 0.01).

**Table 2:** Inhibitory effect of crude ethanol extract (CEE) and BuOH fraction (BuOH fr.) from *S. orientalis* on serum uric acid levels in rats

Group treatment	Number	Dose (mg/kg)	Serum uric acid levels (μmol/L)	Inhibition (%)
Normal	8		114.54 ± 11.23	
Control	8		222.38 ± 24.58^##^	
CEE	8	600	163.58 ± 12.94*	26.4
BuOH fr.	8	120	152.47 ± 11.68**	31.4
Allopurinol	8	10	115.28 ± 11.13**	48.2

Hyperuricemic rats were induced by a potassium oxonate injection 1 h before the last drug administration. The CEE at 600 mg/kg, the BuOH fr. at 120 mg and allopurinol at 10 mg/kg were orally administrated once a day for five consecutive days. The control and normal groups were orally administered with 0.5% CMC-Na. The serum uric acid levels of rats were measured by the phosphotungstic acid method. Values are displayed as mean ± SEM

*p < 0.05, **p< 0.01 vs. hyperuricemic rats (control);

#*p* < 0.05, ##*p* < 0.01 vs. normal rats

### Effect of BuOH fraction on XO activity in rats

**Fig. 1:**
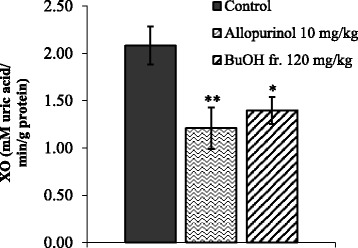
Xanthine oxidase (XO) enzyme from rat liver was treated with the BuOH fraction (BuOH fr.). The activity of XO inhibition was evaluated by monitoring uric acid formation using the spectrophotometric method. Allopurinol was used as a reference agent. Values are presented as mmol/min per g protein and mean ± SEM from 8 animals in the treatment group. * *p* < 0.05, **p < 0.01 vs. control

Detailed investigation for hypouricemic activity of the BuOH fraction was subsequently carried out with in vivo test for liver XO activity in rats. As shown in Fig. 1, the values observed for XO activity in the control and BuOH fr. (120 mg/kg p.o.) groups were 2.08 ± 0.20 and 1.40 ± 0.14 mmol/min per mg protein, respectively. It means that an in vivo inhibition of XO by 32.7% was observed for the BuOH fr. as compared to the control group (*p* < 0.05). In the same experiment, allopurinol inhibited XO activity (41.8% inhibition) at the dose of 10 mg/kg, showing slightly more potent activity than the BuOH fr.

### Effect of BuOH fraction on paw edema in rats

**Fig. 2:**
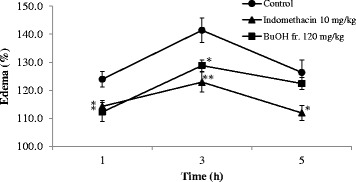
The BuOH fraction (BuOH fr.) was suspended in distilled water and orally administered. Paw edema was induced 30 min after the subplantar injection of 1% (*w*/*v*) carrageenan in buffer saline. The paw edema was measured before (predose) and at intervals of 1, 3, and 5 h after the carrageenan injection. Indomethacin was used as a reference agent. Values are expressed in percent increase of paw before the carrageenan injection and mean ± SEM from 8 animals in the treatment group. * *p* < 0.05, **p < 0.01 vs. control

Anti-inflammatory activity of the BuOH fraction was evaluated using a carrageenan-induced paw edema model. Intraplantar injection of carrageenan (1% *w*/*v*) markedly increased paw volume of the rats, reaching its maximal effect after 3 h (Fig. 2). Treatment with the BuOH fr. (120 mg/kg) reduced the volume of paw edema by 30.4% (*p* < 0.05), as compared to the maximum volume measured before the treatment. Indomethacin (10 mg/kg) served as a positive control, displaying inhibitory activity by reducing 44.6% paw edema.

### Effect of BuOH fraction on mechanical hyperalgesia in rats

**Fig. 3:**
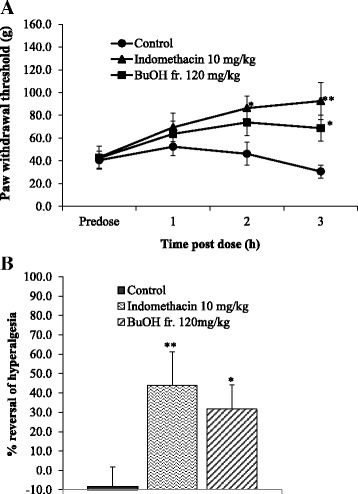
Administration of the BuOH fraction (BuOH fr.) or indomethacin after the treatment with carrageenan significantly reversed mechanical hyperalgesia in rats. Paw withdrawal thresholds were measured before (predose) and at intervals of 1, 3, and 5 h after the carrageenan injection (**a**). Percentage of reversal of hyperalgesia measured 3 h after the vehicle or drug administration (**b**). Values are displayed as mean ± SEM from 8 animals per group. * *p* < 0.05, and ** *p* < 0.01 compared with vehicle

Figure 3 shows the data obtained in rats while mechanical hyperalgesia was induced by carrageenan, which led to the reduction of ipsilateral paw withdrawal thresholds approximately from 150 g to 45–55 g after the injection in naive animals (data not shown). Administration of the BuOH fraction or indomethacin significantly reversed inflammatory mechanical hyperalgesia (Fig. 3a). A maximal 32% reversal was observed 3 h after the administration of the BuOH fr. at dose of 120 mg/kg (*p* < 0.05), while the positive control (indomethacin) produced up to 44% reversal (Fig. 3b).

### Effect of BuOH fraction on thermal hyperalgesia in rats

**Fig. 4:**
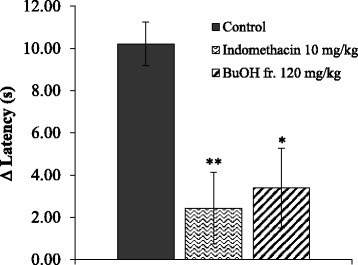
Carrageenan was injected into the hind paw to produce thermal hyperalgesia in rats. The BuOH fraction (BuOH fr.) at the oral dose of 120 mg/kg significantly increased Δ withdrawal latency, inhibiting the inflammatory hyperalgesia response after 3 h of the treatment. Indomethacin (10 mg/kg) was used as a reference compound. Values are expressed in mean ± SEM from 8 animals per group. * *p* < 0.05, **p < 0.01 vs. hyperuricemic rats (control)

Carrageenan injection into the hind paw of rats induced thermal hyperalgesia, exerting maximal effect after 3 h. As shown in Fig. 4, the BuOH fraction remarkably increased Δ withdrawal latency at dose of 120 mg/kg. Indomethacin (10 mg/kg) also exhibited significant anti-nociceptive activity by reducing thermal hyperalgesia.

### Effect of BuOH fraction on synovitis

The inflammatory response in the leg of rats occurred 5 h after intra-synovial injection of sodium urate crystal, and then was gradually increased during subsequent 13 h (data not shown). The hyperalgesic response was stable in all animals for 2 h, prior to the second drug administration. Administration of the BuOH fraction (120 mg/kg, p.o.) alleviated urate-induced synovitis 20 h after injection of a causative inflammatory agent (Table 3). Indomethacin (10 mg/kg, p.o.) also displayed anti-inflammatory effect in this model, resulting in leg tenderness for all animals in the group.

**Table 3:** Effect of BuOH fraction (BuOH fr.) on urate induced synovitis in rats

Group	Number	Dose (mg/kg p.o.)	Score
Control	8	-	3.5 (2–4)
BuOH fr.	8	120	2 (1–3)*
Indomethacin	8	10	1 (1–1)**

Inflammatory responses in the leg receiving sodium urate began 5 h after the injection, and continued to increase during 13 h (data not shown). Hyperalgesic responses were stable in all animals 2 h prior to the second drug administration. An anti-inflammatory agent was effective in this model. Values are expressed in median of score range (min – max). The Kruskal-Wallis test was performed, followed by the Mann-Whitney U test

*p < 0.05, **p < 0.01 vs. control

### Analysis of total phenolic content and identification of phenolic compounds

**Fig. 5:**
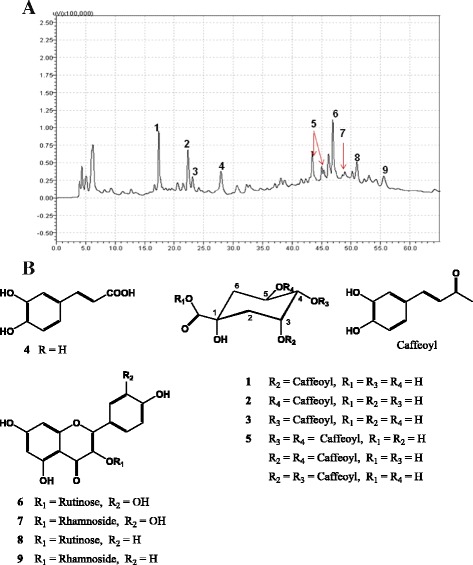
Chromatogram (HPLC-DAD, UV 255 nm) of the BuOH fraction (**a**) and the corresponding chemical structures (**b**)

Major constituents of the CEE were identified as phenolic compounds by analysis of the ^1^H NMR data, and the corresponding signals were also detected in the BuOH fraction--the most active fraction of the extract. Kirenol is an anti-inflammatory diterpene reported previously from this plant [2], but its NMR signals were not present in the BuOH fraction. Therefore, transference of the phenolic compounds found in the CEE into the BuOH fraction was hypothesized to explain the retention of the biological properties. In line with this hypothesis, some phenolic compounds were reported to show anti-gout and anti-inflammatory activities [24, 30–35]. Total phenolic contents for the organic solvent fractions (Hex, EtOAc, and BuOH fractions) and the CEE were quantitatively calculated to be 42.5, 75.7, 173.4, and 138.1 mg/g, respectively. The BuOH fraction, displaying the most potent biological activities, contained the highest total phenolic content, consistent with our hypothesis. The phenolic compounds were isolated by HPLC, and their spectroscopic data (Additional file 1) were compared with those published in the literature [14, 30]: the phenolic compounds were identified as 3-CQA (**1**; 3-caffeoylquinic acid; *t*_R_ = 17.37 min), 5-CQA (**2**; chlorogenic acid; *t*_R_ = 22.32 min), 5-CQA (**3**; 4-caffeoylquinic acid; *t*_R_ = 23.10 min), caffeic acid (**4**, *t*_R_ = 27.92 min), diCQAs (**5**; 3,4-dicaffeoylquinic acid, 3,5-dicaffeoylquinic acid, 3,4-dicaffeoylquinic acid; *t*_R_ = 43.41 min, 45.02 min, and 45.38 min, respectively), rutin (**6**; *t*_R_ = 46.86 min), quercitrin (**7**; *t*_R_ = 49.01 min), kaempferol-3-*O*-rutinoside (**8**; *t*_R_ = 50.94 min), and kaempferol-3-*O*-rhamnoside (**9**; *t*_R_ = 55.63 min) (Fig. 5).

## Discussion

Gout is an emerging and common metabolic disorder closely related to hyperuricemia, the treatment of which aims to relieve acute gouty attacks and to prevent recurrent gouty episodes. Therapeutic approaches for treating gout include applications of anti-inflammatory agents for symptomatic relief, as well as selective inhibition of the terminal steps in uric acid biosynthesis for chronic gout [19]. Combination of the relevant therapies, such as lowering uric acid levels, inhibiting inflammatory responses, and modifying dietary behaviors, was suggested for the treatment of gout [36]. Suppression of XO activity is one of the therapeutic strategies to reduce blood uric acid levels. However, only a few XO inhibitors, e.g., allopurinol and febuxostat, have been clinically used [37]. Although most of the Vietnamese traditional remedies for curing gout disease contain hy-thiem [1, 3], pharmacological research of this medicinal plant associated with the treatment of this metabolic and inflammatory disorder has been underexplored.

We demonstrated in this study that CEE of hy-thiem significantly reduced uric acid levels in oxonate-induced hyperuricemia rats. In addition, in vitro inhibitory activity of the CEE on XO was observed. The most potent activity was detected for the *n*-BuOH-soluble portion. Among the fractions resulting from activity-guided fractionation, the BuOH fraction presented XO inhibitory activity even more potent than that of the whole crude extract (data not shown), and showed no acute and sub-chronic toxicity (Additional file 2). Therefore, transference of active components from the CEE to the BuOH fraction was suggested. The BuOH fraction of hy-thiem showed its hypouricemic effect at dose of 120 mg/kg. Subsequent in vivo studies at the same dose also revealed that the BuOH fraction remarkably inhibited liver XO activity in rats. These observations suggested that the hypouricemic effect of hy-thiem is caused by, at least in part, its inhibitory potency on XO, a key enzyme in the biosynthetic pathway of uric acid. Furthermore, the BuOH fraction also displayed a notable anti-inflammatory and antinociceptive activities in the carrageenan-induced animal model, as shown previously with the crude extract of *S. orientalis* [5, 9]. Finally, deposition of urate crystals, an important initiation factor in the inflammatory process of gout, has been taken into consideration in our experiments. The BuOH fraction was found to have anti-inflammatory effect in the urate-induced synovitis model, which represents acute gouty attacks, confirming that the inhibition of XO is associated with anti-inflammatory responses [37].

Although kirenol was reported to be responsible for the anti-inflammatory and analgesic activities of *S. orientalis* [2], it was absent in the *n*-BuOH-soluble fraction in the present study. Instead, phenolic compounds were identified as major components of the fraction. Therefore, the anti-gout and anti-inflammatory effects of hy-thiem were presumed to be associated with those phenolic compounds in the BuOH fraction [32, 38]. The major constituents in this fraction were identified as caffeic acid analogues (**1–5**) and flavonones (**6–9**). Many studies have showed uricemia lowering and anti-inflammatory activities of caffeic acid analogues [31–34] and flavonones [24, 35], supporting our hypothesis. This is the first report of this plant that phenolic compounds **1–9** are the major constituents, showing anti-inflammatory, analgesic, and anti-gout activities. Of these phenolic compounds, 3-CQA (**1**), chlorogenic acid (**2**), 4-CQA (**3**), di-CQA (**5**), quercitrin (**7**), kaempferol-3-*O*-rutinoside (**8**), and kaempferol-3-*O*-rhamnoside (**9**) were identified for the first time from this species, while a few other phenolic metabolites including caffeic acid (**4**) and rutin (**6**) were reported previously [14].

## Conclusions

This study demonstrates that crude ethanol extract and its BuOH-soluble portion from *S. orientalis* (Vietnamese hy-thiem) show anti-hyperuricemic and anti-inflammatory activities experimentally. The active constituents responsible for the biological activities were identified as phenolic compounds. Our findings support the application of this plant as an indigenous medicine for treating gout, which occurs when metabolic and inflammatory disorders take place together, by its dual pharmacological action.

## Additional files

Additional file 1: Spectroscopic data for phenolic compounds from hythiem (*Siegesbeckia orientalis*). (DOCX 17 kb)

Additional file 2: Report on toxicity study of BuOH fraction. (DOCX 104 kb)

## Abbreviations

BuOH fr.: *n*-BuOH-soluble fraction
CEE: Crude ethanol extract
CMC: Carboxymethylcellulose
CQA: Caffeoylquinic acid
DAD: Diode array detector
HPLC: High performance liquid chromatography
NMR: Nuclear magnetic resonance
XO: Xanthine oxidase
