# Supplementary material for: Anti-Hyperuricemic, Anti-Inflammatory and Analgesic Effects of Siegesbeckia orientalis L. Resulting from the Fraction with High Phenolic Content
Source: BMC Complement Altern Med. 2017 Apr 4;17:191. doi: 10.1186/s12906-017-1698-z (PMC5379685; doi:10.1186/s12906-017-1698-z)
Supplement: Supplementary file 1 — Spectroscopic data for phenolic compounds from hythiem (Siegesbeckia orientalis). (DOCX 17 kb) [file 12906_2017_1698_MOESM1_ESM.docx]

**SPECTROSCOPIC DATA FOR PHENOLIC COMPOUNDS FROM HYTHIEM (*SIEGESBECKIA ORIENTALIS*)**

Compound **1** (3-Caffeoyl quinic acid): UV (MeOH) λ_max_ 218, 235, 329 nm. ^1^H-NMR (300MHz, CD_3_OD) δ_H_ (ppm): 2.03 – 2.38 (4H, m, H-2, 6), 4.31 (1H, m, H-3), 5.03 (1H, m, H-4), 5.73 (1H, m, H-5), 6.63-7.10 (3H, m, H-2′, H-5′, H-6′), 6.28 (1H, d, *J* = 16.0 Hz, H-7′), 7.57 (1H, d, *J* = 16.0 Hz, H-8′).

Compound **2** (Chlorogenic acid): UV (MeOH) λ_max_ 219, 244, 329 nm; ^1^H-NMR (500MHz, CD_3_OD) δ_H_ (ppm): 2.06 – 2.12 (2H, m, H-2), 4.19 (1H, m, H-3), 3.75 (1H, m, H-4), 2.18 – 2.27 (2H, m, H-6), 5.35 (1H, m, H-5), 7.07 (1H, d, *J* = 2.0 Hz, H-2′), 6.79 (1H, d, *J* = 8.0 Hz, H-5′), 6.97 (1H, dd, *J* = 8.5, 2.0 Hz, H-6′), 6.28 (1H, d, *J* = 16.0 Hz, H-7′), 7.57 (1H, d, *J* = 16.0 Hz, H-8′); APCI-MS: *m/z* 353 [M - H]^−^ (negative mode).

Compound **3** (4-Caffeoyl quinic acid): UV (MeOH) λ_max_ 217, 244, 328 nm.

Compound **4** (Caffeic acid): UV (MeOH) λ_max_ 219, 233, 294, 321 nm; ^1^H-NMR (500 MHz, DMSO-*d_6_*) δ_H_ (ppm): 6.39 (1H, d, *J* = 2.0 Hz, H-2), 6.87 (1H, d, *J* = 8.5 Hz, H-5), 7.53 (1H, dd, *J* = 8.5, 2.0 Hz, H-6); 6.17 (1H, d, *J* = 16.0 Hz, H-7), 7.66 (1H, d, *J* = 16.0 Hz, H-8); ESI-MS: *m/z* 302 [M]^+^.

Compound **5 (1)** (diCaffeoyl quinic acid): UV (MeOH) λ_max_ 218, 244, 300, 329 nm; ^1^H-NMR (300MHz, CD_3_OD) δ_H_ (ppm): 2.01 – 2.23 (4H, m, H-2 & H-6), 4.34 (1H, m, H-3), 5.09 (1H, m, H-4), 5.59 (1H, m, H-5), 7.07 (1H, d, *J* = 2.0 Hz, H-2′), 6.79 (1H, d, *J* = 8.0 Hz, H-5′), 6.97 (1H, dd, *J* = 8.5, 2.0 Hz, H-6′), 6.23 (2H, d, *J* = 16.0 Hz, H-7′), 7.57 (2H, d, *J* = 16.0 Hz, H-8′ and H-8′′).

Compound **5(2)** (diCaffeoyl quinic acid): UV (MeOH) λ_max_ 218.5, 235.5, 244.5, 329.5, 329 nm; ^1^H-NMR (300MHz, CD_3_OD) δ_H_ (ppm): 2.18 – 2.33 (4H, m, H-2 & H-6), 4.00 (1H, m, H-3), 4.96 (1H, overlap, H-4), 5.42 (1H, m, H-5), 6.76-7.06 (6H, m, H-2′, H-5′, H-6′, H-2′′, H-5′′ and H-6′′), 6.28 (2H, d, *J* = 16.0 Hz, H-7′ and 7′′), 7.57 (2H, d, *J* = 16.0 Hz, H-8′ and H-8′′).

Compound **6** (Rutin): UV (MeOH) λ_max_ 255, 289 (sh), 372 nm; ^1^H-NMR (DMSO-*d_6_*) δ_H_ (ppm): 6.17 (1H, d, *J* = 2.0 Hz, H-6), 6.39 (1H, d, *J* = 2.0 Hz, H-8), 7.66 (1H, d, *J* = 2.0 Hz, H-2′), 6.87 (1H, d, *J* = 8.5 Hz, H-5′), 7.53 (1H, dd, *J* = 8.5, 2.0 Hz, H-6′); ESI-MS: *m/z* 302 [M]^+^, 301 [M - H]^−^ (negative mode).

Compound **7** (Quercitrin): UV (MeOH) λ_max_ 252, 293 (sh), 348 nm; ^1^H-NMR (DMSO-*d_6_*) δ_H_ (ppm): 6.11 (1H, d, *J* = 2.0 Hz, H-6), 6.27 (1H, d, *J* = 2.0 Hz, H-8), 7.24 (1H, d, *J* = 2.0 Hz, H-2′), 6.82 (1H, d, *J* = 8.0 Hz, H-5′), 7.21 (1H, dd, *J* = 8.0, 2.0 Hz, H-6′), 5.25 (1H, d, *J* = 1.5 Hz, H-1′′), 4.13 (1H, dd, *J* = 1.5, 3.0 Hz, H-2′′), 3.66 (1H, dd, *J* = 3.5, 9.5 Hz, H-3′′), 3.32 (1H, m H-4′′), 3.25 (1H, t, *J* = 6.5 Hz, H-5′′), 0.85 (1H, d, *J* = 6.5 Hz, H-6′′); ESI-MS: *m/z* 448 [M]^+^, 447 [M - H]^−^ (negative mode).

Compound **8** (Kaempferol-3-*O*-rutinoside): UV (MeOH) λ_max_ 266, 364 nm;

Compound **9** (Kaempferol-3-*O*-*α*-L-rhamnopyranoside): UV (MeOH) λ_max_ 264, 280 (sh), 341 nm; ^1^H-NMR (DMSO-*d_6_*) δ_H_ (ppm): 6.11 (1H, d, *J* = 2.0 Hz, H-6), 6.27 (1H, d, *J* = 2.0 Hz, H-8), 7.24 (1H, d, *J* = 2.0 Hz, H-2′), 6.82 (1H, d, *J* = 8.0 Hz, H-5′), 7.21 (1H, dd, *J* = 8.0, 2.0 Hz, H-6′), 5.25 (1H, d, *J* = 1.5 Hz, H-1′′), 4.13 (1H, dd, *J* = 1.5, 3.0 Hz, H-2′′), 3.66 (1H, dd, *J* = 3.5, 9.5 Hz, H-3′′), 3.32 (1H, m H-4′′), 3.25 (1H, t, *J* = 6.5 Hz, H-5′′), 0.85 (1H, d, *J* = 6.5 Hz, H-6′′); ESI-MS: *m/z* 432 [M]^+^, 431 [M - H]^−^ (negative mode).
