# Supplementary material for: Anti-Hyperuricemic, Anti-Inflammatory and Analgesic Effects of Siegesbeckia orientalis L. Resulting from the Fraction with High Phenolic Content
Source: BMC Complement Altern Med. 2017 Apr 4;17:191. doi: 10.1186/s12906-017-1698-z (PMC5379685; doi:10.1186/s12906-017-1698-z)
Supplement: Supplementary file 2 — Report on toxicity study of BuOH fraction. (DOCX 104 kb) [file 12906_2017_1698_MOESM2_ESM.docx]

**REPORT ON TOXICITY STUDY OF BuOH FRACTION**

**I. STUDY ON ACUTE TOXICITY**

**1. Method**

The acute toxicity study was implemented in compliance with Guidelines of OECD and Vietnam’s Ministry of Health.

***1.1. Study design***

*Administration of doses*

Animals were fasted prior to dosing (food but not water was withheld for 2-3 hours). Following the period of fasting, body weight of each animal was measured and the dose was calculated based on the body weight. Test product was administered in a twice divided dose by gavage using intubation needle. Dosing interval did not exceed 24 hours but was not less than 2 hours. The volume of test product delivered was 0.2 mL once per 10 g of body weight. After the product had been administered, food was withheld for a further 2 hours.

*Sighting test*

The purpose of the sighting study is to allow selection of the appropriate starting dose for the main test. The sighting study is completed when a decision on the starting dose for the main study can be made (or if a death is seen at the lowest fixed dose). A period of at least 24 hours will be allowed between the dosing of each animal. All animals will be observed for at least 14 days.

*Main test*

Mice were randomly divided into 10-individual groups. Doses of test product in main test were inferred from result of sighting test. As a rule, the chosen doses were between maximum dose, which did not kill any animal, and minimum dose which killed all of animals. Animals were observed for a total of 14 days (with special attention given during the first 4 hours).

***1.2. Observations***

Animals were observed individually after dosing at least once during the first 30 minutes, periodically during the first 24 hours, with special attention given during the first 4 hours, and daily thereafter, for 72 hours, except where they need to be removed from the study and humanely killed for animal welfare reasons or were found dead. Observation was taken until 14 days after dosing.

All observations were systematically recorded, with individual records being maintained for each animal during the first 72 after dosing:

- General signs: somatomotor activity and behaviour pattern, changes in skin, fur, eyes, mucous membranes, feces and urine…

- Water and food consumption.

- The number of deaths: The percentage of animals killed by each dose level was recorded to estimate LD_50_.

- All dead animals were subjected to gross necropsy as soon as possible and pathological changes should be recorded.

- Additional observations were taken until 14 days after dosing.

***1.3. Statistical analysis***

- LD_50_ (if available) was estimated by probit analysis using SPSS 16.0.

**2. Results**

***2.1. Sighting test***

10 mice were separated into groups of 2 animals used test product with gradually increased doses:

- No abnormal change was observed during 4 hours after dosing. All mice survived until completion a 14-day observational course.

- Results of sighting test suggested that dose of 2000 mg per kg b.w administered in a total volume of 0.4 mL/10 g b.w was maximum dose which can be prepared to use in mice by gavage administration; no any death was observed in mice exposed to this dose.

***2.2. Main test***

Taking into account the results of sighting test, a main test was performed in 10 healthy female mice. Dosage regimen for this group was as following:

**Table 1.** Dosing design for acute oral toxicity study of BuOH fr.

| Group | Number of mice | Dose  (mg/kg b.w) | Gavage volume  (mL/10g b.w) | The number of doses |
| --- | --- | --- | --- | --- |
| 1 | 10 | 2000 | 0.2 | 2 |

- At 4 hours after dosing: no abnormality with respect to food and water consumption, somatomotor activity and behaviour pattern, reflex, no changes in skin and fur, eyes and mucous membranes, feces and urine excretion, and also respiratory status was observed in both groups.

- No deaths occurred during the 72h- procedure and clinical observations did not show evidences of drug-related toxicity.

- 100% animals of each group survived until 14^th^ day of the study. As a result, LD_50_ value was unknown.

**3. Conclusion**

At oral dose of 2000 mg/kg b.w (the maximum dose that can be prepared to use in mice by gavage administration), BuOH fr. did not induce deaths or toxic symptoms in acute toxicity study.

**II.** **STUDY ON SUB-CHRONIC TOXICITY**

**1. Method**

The sub-chronic toxicity study was performed to the OECD TG 407 protocol (Guideline for Repeated Dose 28-day Oral Toxicity Study in Rodents).

***1.1. Study design***

Rats were randomly divided into three groups for each sex (male or female): two groups treated with 2 levels of BuOH fr. by gavage at dose 360 mg/kg, 1290 mg/kg and a control group dosed by the same route with the vehicle (ultrapure water) alone.

The animals were dosed with the test product or vehicle daily at 9 am for a period of 28 days. After that, if it was considered to be necessary, all rats were housed for another 2-week period in which no any treatment was applied and recovery of organs and physiological functions was observed. General observations were made daily during the study and animal body weight which also served as the basis for dose adjustment was recorded weekly. At the end of the study (after 28 days or 28 days plus 2 weeks of recovery), blood was collected from the retro-orbital venous plexus of all animals into two tubes: one containing anticoagulants for immediate analysis of haematological parameters and the other to separate serum for biochemical estimations. All animals were subjected to gross pathological examination. Hepatic and renal samples of animals selected randomly from each group were preserved for further histopathological examination.

***1.2. Items of observation***

- Observation on general condition: general clinical observations were made at least once a day, at the same time each day. Signs noted specially include, but not be limited to, changes in skin, fur, eyes, mucous membranes, occurrence of secretions and excretions and autonomic activity (e.g., lacrimation, pilo-erection, pupil size, unusual respiratory pattern), water and food consumption.

- Animal body weight which also served as the basis for dose adjustment was recorded weekly.

- Hematology: the hematological parameters hemoglobin concentra-tion (Hb), mean corpuscular volume (MCV), total erythrocyte count (RBC), hematocrit (HCT), total and differential leukocyte count, platelet count (PLT) were determined by standard methods using URIT-3000 VET Plus automated hematology analyzer.

- Clinical biochemistry: serum concentrations of glucose, total cholesterol, creatinine and albumin were analyzed using TC 3300 plus (Teco Diagnostics, USA). The serum alanine aminotransferase (ALT), aspartate aminotransferase (AST) were also assayed using TC 3300 plus (Teco Diagnostics, USA).

- Gross pathology: liver, kidneys, adrenals, spleen and heart of all animals were trimmed of any adherent tissue, as appropriate, and their wet weight, also expressed as ratios of them to body weight, taken as soon as possible after dissection to avoid drying.

- Histopathology: tissue samples were preserved in 10% neutral buffered formalin. Tissues from liver, kidneys were immersed in 10% Carnoy solution, then embedded in paraffin and cut with a microtome set at 5 - 7 μm. The sections were stained with hematoxylin and eosin and examined by light microscopy.

***1.3. Statistical analysis***

Sexes were analysed separately. All data are expressed as mean ± standard error of the mean. Comparisons between groups were performed by analysis of variance (ANOVA). The differences were considered to be significant if p < 0.05.

**2. Results**

***2.1. The effects of BuOH fr. on general status in rats***

It was observed that the animals treated with BuOH fr. were healthy. No unusual changes in behavior or in locomotor activity, no ataxia, and no signs of intoxication were observed during the 28-day period. No differences were found in growth between the control group and the animals treated with different levels of BuOH fr. (Figures 1 and 2). The food and water consumption of male and female rats of control and experimental groups was similar.


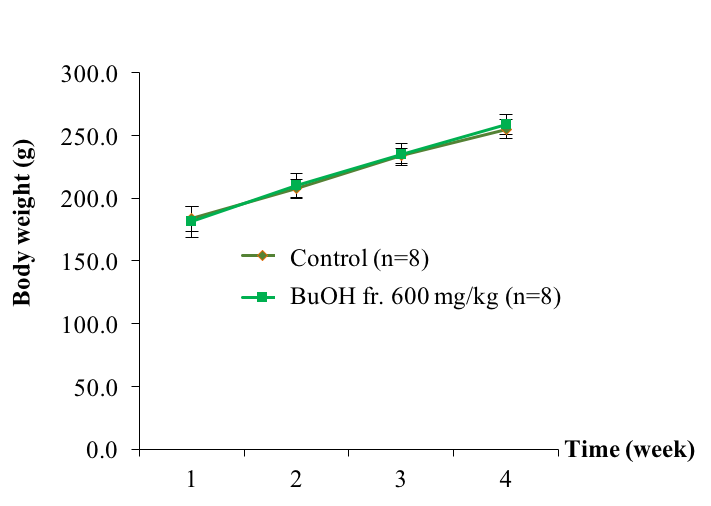


**Figure 1.** Growth curve of male rats administered BuOH fr.


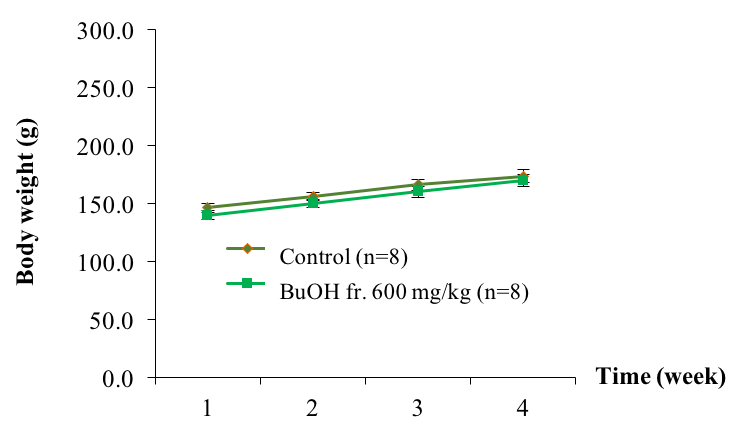


**Figure 2.** Growth curve of female rats administered BuOH fr.

***2.2 The effects of BuOH fr. on hematological parameters in rats***

There was not any significant difference in terms of the hematological values observed between control and treated groups (Table 2).

**Table 2.** Hematological data of rats administered with BuOH fr. for 28 days

| Hematological data | Male | | | Female | |
| --- | --- | --- | --- | --- | --- |
|  | Control (n=8) | BuOH fr. 600 mg/kg (n=8) | Control (n=8) | | BuOH fr. 600 mg/kg (n=8) |
| RBC (10^12^/L) | 9,06 ± 0,66 | 8,98 ± 0,21 | 8,38 ± 0,31 | | 8,23 ± 0,19 |
| HGB (g/dL) | 14,5 ± 1,0 | 14,5 ± 0,5 | 13,6 ± 0,6 | | 13,4 ± 0,4 |
| HCT (%) | 51,6 ± 4,1 | 51,8 ± 1,4 | 48,3 ± 1,9 | | 46,1 ± 1,5 |
| MCV (fL) | 56,6 ± 1,0 | 57,7 ± 0,7 | 57,6 ± 0,4 | | 55,9 ± 0,8 |
| MCH (pg) | 16,1 ± 0,2 | 16,1 ± 0,3 | 16,2 ± 0,2 | | 16,3 ± 0,2 |
| MCHC (g/dL) | 28,4 ± 0,5 | 28,0 ± 0,5 | 28,2 ± 0,3 | | 28,8 ± 0,2 |
| PLT (10^9^/L) | 832,5 ± 81,9 | 927,4 ± 68,1 | 796,4 ± 41,5 | | 914,6 ± 36,4 |
| WBC (10^9^/L) | 12,3 ± 1,4 | 11,4 ± 0,8 | 14,5 ± 1,0 | | 12,9 ± 0,7 |
| LYM% (%) | 59,8 ± 5,3 | 59,1 ± 1,7 | 69,6 ± 3,2 | | 65,1 ± 2,6 |

***2.3. The effects of BuOH fr. on some biochemical parameters in rats***

There was not any significant difference in terms of AST, ALT, creatinine, total cholesterol, total protein, glucose level observed between control and treated groups (Table 3).

**Table 3.** Biochemical parameters of rats administered with BuOH fr. for 28 days

| Biochemical parameters | Male | | Female | |
| --- | --- | --- | --- | --- |
|  | Control (n=8) | BuOH fr. 600 mg/kg (n=8) | Control (n=8) | BuOH fr. 600 mg/kg (n=8) |
| AST (U/L) | 24,0 ± 4,5 | 24,8 ± 3,0 | 17,4 ± 1,1 | 17,2 ± 1,7 |
| ALT (U/L) | 31,1 ± 3,4 | 35,7 ± 6,0 | 26,7 ± 2,9 | 22,8 ± 3,7 |
| Glucose (mmol/L) | 3,6 ± 0,2 | 3,7 ± 0,2 | 6,3 ± 0,7 | 6,3 ± 0,5 |
| Cholesterol (mmol/L) | 1,3 ± 0,1 | 1,4 ± 0,1 | 1,3 ± 0,1 | 1,3 ± 0,1 |
| Protein (g/L) | 6,1 ± 0,3 | 5,4 ± 0,2^*^ | 6,3 ± 0,1 | 6,0 ± 0,2 |
| Creatinin (μmol/L) | 224,0 ± 17,6 | 291,7 ± 25,0 | 283,0 ± 17,9 | 239,0 ± 16,7 |

***2.4. The effects of BuOH fr. on histopathological data in rat***

*Gross pathology*

Table 4 depicts the organ-to-body mass ratios of animals at the end of 28 days’ administration. No abnormal changes were observed in organ mass with respect to body mass of BuOH fr. 600 mg/kg treated rats in comparison with controls. Observations of gross pathology immediately after dissection, on rats of all groups were found to be uniformly healthy, lacking in any apparent pathological abnormalities.

**Table 4.** Percentage organ weight to body mass of rats administered with BuOH fr.

| Organ-to-body weight ratio (%) | Male | | Female | |
| --- | --- | --- | --- | --- |
|  | Control (n=8) | BuOH fr. 600 mg/kg (n=8) | Control (n=8) | BuOH fr. 600 mg/kg (n=8) |
| Heart | 0,58 ± 0,06 | 0,54 ± 0,04 | 0,70 ± 0,07 | 0,68 ± 0,08 |
| Liver | 4,22 ± 0,30 | 4,35 ± 0,19 | 5,83 ± 0,36 | 5,80 ± 0,40 |
| Kidney | 0,71 ± 0,03 | 0,74 ± 0,01 | 0,86 ± 0,06 | 0,95 ± 0,06 |
| Adrenal gland | 0.015± 0.001 | 0.015 ± 0.001 | 0.029 ± 0.003 | 0.031± 0.003 |
| Spleen | 0,23 ± 0,01 | 0,25 ± 0,02 | 0,33 ± 0,02 | 0,36 ± 0,03 |

*Microscopic histopathology*

Microscopic examination of hepatic and renal tissues taken from the control and BuOH fr. 600 mg/kg treated animals did not show any treatment-related differences. The incidence of histopathological findings was similar in treated and control animals (data not shown). None of these abnormalities could, therefore, be attributed to the treatment with BuOH fr. 600 mg/kg.

**3. Conclusion**

In conclusion, based on the present results, the high oral dose of BuOH fr. 600 mg/kg investigated in the 28 days repeated doses scheme did not show any difference among controls and treated animals, thus representing a dose level of non-observable toxic effects (NOEL). Due to the lack of any adverse effects apparent during this study, the no observed-adverse- effect level (NOAEL) was set at the high dose of 600 mg/kg/day BuOH fr. Overall, no treatment-related toxicity has been observed after 28 days.
